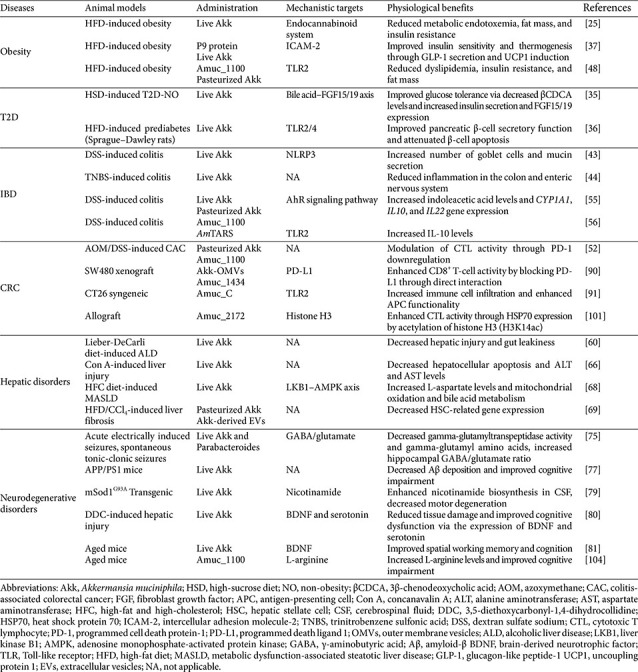# Unveiling the Molecular Repertoire of *Akkermansia muciniphila*: From Mechanistic Insights to Precision Biotherapeutics

**DOI:** 10.4014/jmb.2604.04017

**Published:** 2026-06-01

**Authors:** Tae-Hwan Kim, Su-Man Kim, Myung Hee Kim

**Affiliations:** 1 College of Veterinary Medicine, Chungnam National University, Daejeon 34134, Republic of Korea; 2 Department of Biology Education, Chonnam National University, Gwangju 61186, Republic of Korea; 3 Microbiome Convergence Research Center, Korea Research Institute of Bioscience and Biotechnology (KRIBB), Daejeon 34141, Republic of Korea

**Keywords:** *Akkermansia muciniphila*, Host-microbe crosstalk, Next-generation probiotic, Therapeutic effectors, Immune homeostasis

## Abstract

The gut microbiota has been established as a cornerstone of host physiological homeostasis. Among its diverse members, *Akkermansia muciniphila* has gained significant prominence as a premier next-generation probiotic candidate, demonstrating broad efficacy in alleviating metabolic disorders, inflammatory bowel disease, and neurodegenerative conditions. While early research primarily focused on clinical correlations and population abundance, recent scientific paradigms have shifted toward elucidating the underlying molecular mechanisms and specific host-microbe interactions. This review provides a comprehensive synthesis of recent breakthroughs in identifying *A. muciniphila*-derived bioactive effectors, including structural components, secreted enzymes, and signaling peptides. We examine how these molecular postbiotics orchestrate host health by reinforcing intestinal barrier integrity, modulating systemic immune responses, and reprogramming the tumor microenvironment. By integrating these multifaceted modes of action into a unified framework, we evaluate the therapeutic potential of both live bacteria and cell-free derivatives. Furthermore, we address the critical challenges regarding strain-specific efficacy and ecological impacts, providing a strategic roadmap for the clinical translation of *A. muciniphila* into the next frontier of precision microbiome medicine.

## Introduction

The human gastrointestinal tract harbors a complex and dynamic consortium of microorganisms, collectively termed the gut microbiota. This ecosystem, comprising bacteria (predominant), viruses, and fungi, plays an indispensable role in maintaining host physiological homeostasis. The gut microbiome–referring to the microbiota and their genetic material–perform a vast array of metabolic functions that complement host biology. Importantly, a resilient and diverse microbial composition is fundamental to host health, providing a protective barrier against metabolic dysregulation, chronic inflammation, and pathogen colonization [1-6]. Consequently, deciphering the intricate cross-talk between the gut microbiota and host has become a frontier in developing precision biotherapeutics for previously intractable diseases.

The integration of high-throughput shotgun metagenomic sequencing and multi-omics has fundamentally revolutionized our understanding of the microbiota-host axis, particularly by elucidating the complex associations between the microbiota and diverse pathologies, such as obesity, type 2 diabetes (T2D), and inflammatory bowel disease (IBD) [7]. Accumulating evidence has demonstrated that the enrichment of classical beneficial bacteria, notably *Lactobacillus* and *Bifidobacterium*, plays a pivotal role in promoting gut health [8, 9]. Traditionally, the *Firmicutes*/*Bacteroidetes* (F/B) ratio is employed as a key indicator of health status, as shifts in this ratio reflect the collective microbial capacity to ferment dietary compounds into short-chain fatty acids (SCFAs) [10, 11].

However, recent scientific paradigms have transitioned from these broad microbial landscapes toward the identification of specific keystone species with potent therapeutic potential. Notably, *Akkermansia muciniphila* has emerged as a premier candidate in this regard. Since it was first reported to exert clinically relevant beneficial effects on metabolic health, two decades of extensive research has substantiated its ameliorative effect against a spectrum of metabolic and inflammatory disorders [12-14].

*A. muciniphila* is a Gram-negative, strictly anaerobic bacterium belonging to the phylum *Verrucomicrobia*, a taxon containing relatively few identified species, and was first isolated from human feces in 2004 [15]. This bacterium primarily localizes to the distal small intestine and colon, where it uniquely thrives by utilizing host-derived mucin as its sole source of carbon and nitrogen [15-17]. The apical surface of the intestinal epithelium is shielded by the mucus layer—a complex network of heavily glycosylated proteins (mucins) rich in serine and threonine, secreted by goblet cells. This specialized niche facilitates *A. muciniphila* colonization and enables the bacterium to modulate mucosal immune responses and promote tolerance to commensal microbiota [18-20]. Although *A. muciniphila* typically accounts for 1–4% of the total gut microbiota in healthy adults, its abundance serves as a crucial biomarker of gut homeostasis and is strongly correlated with improved metabolic phenotypes [21, 22].

In this review, we provide a comprehensive synthesis of the compelling evidence linking *A. muciniphila* to host health. Furthermore, we highlight its therapeutic potential as a next-generation probiotic (NGP) by elucidating the molecular mechanisms underlying *A. muciniphila*-derived bioactive molecules and their implications for future clinical applications.

## Physiological Roles of *A. muciniphila* in Host Health

Accumulating evidence from preclinical studies has highlighted the multifaceted roles of *A. muciniphila* in orchestrating host physiological responses across a diverse array of pathological conditions. By employing various animal models, researchers have identified specific molecular pathways and signaling cascades through which this bacterium exerts its therapeutic and homeostatic effects. The physiological functions and underlying mechanisms of *A. muciniphila* across these disease models are systematically summarized in Table 1. Below, we provide a comprehensive discussion regarding its efficacy and distinct modes of action—ranging from metabolic regulation to immune modulation—for each disease category.

### Metabolic Homeostasis and Disorders

As the global prevalence of obesity reaches critical levels, the gut microbiota has emerged as a central regulator of host energy metabolism and promising therapeutic target. Early landmark studies demonstrated that fecal microbiota transplantation (FMT) from lean donors to obese recipients significantly ameliorated metabolic dysfunction, establishing a causal link between gut microbes and obesity pathogenesis [23]. Subsequent research confirmed that diet-induced obesity drastically shifts microbial topography, triggering systemic inflammatory and metabolic reprogramming [24, 25]. Notably, this dysbiosis is often reversible through targeted dietary interventions or probiotic supplementation [26, 27].

One of the key mechanisms linking the microbiota to adipose tissue physiology involves the lipopolysaccharide (LPS)-endocannabinoid system-regulatory loop. Microbiota-derived LPS modulates adipose metabolism by interfering with cannabinoid-driven adipogenesis, thereby promoting fat storage and chronic low-grade inflammation during obesity [28]. In this context, Everard *et al*. [25] identified *A. muciniphila* as a crucial metabolic gatekeeper: its abundance was markedly reduced in obese mice, while restoration of its levels via prebiotic administration significantly improved the metabolic profile. Although early reports suggested that only viable *A. muciniphila* could reverse high-fat diet (HFD)-induced metabolic disorders, the strong inverse correlation between *A. muciniphila* abundance and inflammatory markers suggests that this bacterium plays a fundamental role in maintaining adipose tissue homeostasis [29-31].

T2D is a multifaceted metabolic disorder characterized by hyperglycemia resulting from progressive insulin resistance or pancreatic β-cell dysfunction [32, 33]. While metagenome-wide association studies initially revealed a moderate degree of gut dysbiosis in patients with T2D [1], the specific role of *A. muciniphila* is unclear and thus has been a subject of intense investigation. Although clinical reports on its abundance in T2D cohorts have occasionally been inconsistent [34], an overwhelming majority of studies indicate a significant decrease in *A. muciniphila* in diabetic states, which correlates with impaired insulin secretion and glucose intolerance [35].

Mechanistically, *A. muciniphila* reputedly augments pancreatic β-cell function, enhancing insulin secretion and attenuating apoptosis by downregulating Toll-like receptor (TLR) signaling pathways in prediabetic models [36]. A pivotal study recently identified a specific 84 kDa protein secreted by *A. muciniphila*, designated P9. This bioactive molecule interacts with intercellular adhesion molecule-2 (ICAM-2) to induce the secretion of glucagon-like peptide-1 (GLP-1) and enhance thermogenesis [37]. Overall, these findings position *A. muciniphila* not merely as a passive commensal, but as an active endocrine modulator capable of restoring glucose homeostasis through sophisticated molecular signaling cascades.

### Intestinal Barrier Integrity and IBDs

IBD, comprising Crohn’s disease and ulcerative colitis, is a chronic inflammatory disorder characterized by a dysregulated immune response to the intestinal microbiota [2, 38]. Given the central role of dysbiosis in IBD pathogenesis, therapeutic strategies have increasingly focused on modulating the gut ecosystem through supplementation with specific bacteria to restore immune homeostasis [14]. Clinical and preclinical evidence has consistently highlighted a significant reduction in *A. muciniphila* abundance in both patients with IBD and dextran sulfate sodium (DSS)- or trinitrobenzene sulfonic acid-induced murine colitis models [39-44]. Consequently, *A. muciniphila* has gained considerable attention as a potent therapeutic target due to its dual role in reinforcing gut barrier integrity and orchestrating host immune responses [42, 45].

The intestinal mucosal barrier, which serves as the primary defense against luminal antigens and pathogens, is frequently compromised in IBD. This impairment allows direct translocation of commensal bacteria and pathogens to the underlying epithelial cells, triggering a cascade of pro-inflammatory signals and leaky gut syndrome [46]. *A. muciniphila* counteracts this process by stimulating goblet cell differentiation and enhancing the biosynthesis of essential mucins [47]. Recent studies have confirmed that *A. muciniphila* administration upregulates the expression of key mucin genes (including *Muc1*, *Muc2*, *Muc5*, and *Muc13*) and tight junction proteins, such as occludin, claudins, and zonula occludens-1 [25, 48]. By fortifying these physical barriers, *A. muciniphila* effectively sequesters luminal antigens from the host immune system.

Despite these protective roles, *A. muciniphila* exhibits threonine auxotrophy and therefore produces a range of mucin-degrading enzymes to utilize mucin as a nutrient source, raising concerns that its expansion under certain conditions may compromise the integrity of the mucus layer [49]. This dual biological nature of *A. muciniphila* appears to be influenced by multiple factors including host diet, baseline microbiota composition, and inflammatory status indicating that its net effect may be context dependent rather than uniformly detrimental [50, 51]. Thus, *A. muciniphila* should be understood as part of a dynamic host–microbe interaction that may ultimately enhance mucosal resilience when present at physiological levels.

The immunomodulatory capacity of *A. muciniphila* extends beyond physical barrier reinforcement. In the context of IBD, it suppresses the mucosal infiltration of macrophages and cytotoxic T lymphocytes (CTLs), thereby attenuating colonic inflammation [52]. In particular, *A. muciniphila* promotes the expansion of regulatory T cells (Tregs) and stimulates interleukin (IL)-22 secretion by type 3 innate lymphoid cells, which are critical for mucosal repair and epithelial regeneration [52, 53]. Simultaneously, it downregulates the production of key pro-inflammatory cytokines, including tumor necrosis factor (TNF)-α, IL-1β, IL-6, IL-18, and IL-33, while fostering an anti-inflammatory milieu characterized by elevated IL-10 levels [54, 55].

Significantly, recent investigations have broadened the therapeutic scope of *A. muciniphila* by demonstrating that its beneficial effects are not conferred by only viable cells. Pasteurized *A. muciniphila*, its extracellular vesicles (EVs), and specific derived molecules— most notably the outer membrane protein Amuc_1100—can partially or fully recapitulate the anti-inflammatory effects observed with the live bacteria [42, 49, 52, 56]. For instance, *A. muciniphila*-derived EVs facilitate the delivery of bioactive cargo to deeper mucosal layers, offering a safer and more stable alternative to live biotherapeutics. These findings underscore the therapeutic versatility of *A. muciniphila* and support the development of standardized, non-viable, microbiome-derived pharmacological interventions for IBD.

### Modulation of the Gut-Liver Axis in Hepatic Disorders

The portal vein serves as a critical anatomical conduit for the bidirectional gut-liver axis, channeling intestinal blood—laden with diverse metabolites, dietary constituents, and microbiota-derived molecular patterns, such as LPS, trimethylamine N-oxide, and SCFAs—directly to the liver. Reciprocally, the liver modulates intestinal homeostasis through the biliary secretion of bile acids, which exert potent antimicrobial and metabolic signaling effects [57]. This intricate cross-talk implies that intestinal dysbiosis can fundamentally undermine hepatic integrity, a premise supported by the involvement of microbial imbalances in the pathogenesis of various hepatopathies [3, 5].

Specifically, metabolic dysfunction-associated steatotic liver disease (MASLD)—formerly termed non-alcoholic fatty liver disease—and alcoholic liver disease are hallmark conditions characterized by a compromised intestinal barrier and a distinctive microbial dysbiosis [58-60]. Clinical and preclinical evidence consistently indicates Proteobacteria enrichment in patients with steatosis and metabolic dysfunction-associated steatohepatitis, while a marked depletion of *Faecalibacterium prausnitzii* is frequently observed in cirrhotic cohorts [5]. This pathological framework is further supported by the positive correlation between increased intestinal permeability (leaky gut) and the translocation of gut-derived inflammatory mediators, which serve as potent drivers of MASLD progression [61-64]. The causal involvement of the microbiota in hepatic pathology is explicitly evidenced by studies in germ-free mice, demonstrating that microbial composition dictates susceptibility to Fas-induced liver injury via myeloid differentiation primary response 88-dependent signaling [61]. Consequently, the preservation of a homeostatic gut-immune axis by the microbiota is a critical determinant of hepatic disease control [65].

While research on *A. muciniphila* in the context of hepatic disorders is relatively nascent compared with that on obesity or IBD, emerging evidence has highlighted its potent hepatoprotective properties. In concanavalin A-induced models of acute liver injury, *A. muciniphila* administration attenuates hepatocyte apoptosis and systemic pro-inflammatory cytokine surges [66]. Moreover, in chronic metabolic models, it ameliorates HFD-induced hepatic steatosis by suppressing *de novo* lipogenesis and downregulating inflammatory cascades [67].

A key mechanistic breakthrough recently revealed that *A. muciniphila* facilitates the transport of L-aspartate from the gut to the liver, where it activates the liver kinase B1– adenosine monophosphate (AMP)-activated protein kinase (AMPK) axis. This activation promotes hepatic lipid oxidation and mitigates metabolic dysfunction [68]. Furthermore, both viable and pasteurized *A. muciniphila*, as well as its EVs, fortify the intestinal mucosal barrier, thereby preventing the translocation of endotoxins that drive HFD/CCl_4_-induced injury and ethanol-induced liver damage [60, 69]. Beyond structural reinforcement, *A. muciniphila* supplementation has demonstrated efficacy in reducing liver fibrosis and hyperammonemia, likely by restructuring the small intestinal microbiota and subsequently alleviating hepatic encephalopathy (HE). Collectively, these findings position *A. muciniphila* as a promising therapeutic agent for modulating the gut-liver axis and treating complex hepatic pathologies.

### Neuro-Immunological Crosstalk in Neurodegenerative Conditions

The gut-brain axis represents a sophisticated bidirectional communication network integrating the gastrointestinal tract and central nervous system (CNS). This inter-organ dialogue is mediated through an array of signaling conduits, including the vagus nerve, neuroendocrine hormones, microbial metabolites, and systemic immunological cascades [70]. The gut microbiota serves as a pivotal rheostat in this axis, modulating neurological processes and cognitive functions by producing neurotransmitters, SCFAs, and various immunomodulatory molecules [71]. Consequently, the dysregulation of the gut-brain axis has been implicated in a spectrum of neuropsychiatric and neurodegenerative conditions, ranging from depression to complex neurodegenerative diseases such as Alzheimer’s disease (AD) [72-74].

Emerging evidence has suggested that *A. muciniphila* may function as a key microbial modulator in the pathogenesis of neurodegenerative disorders, including AD, autism spectrum disorder (ASD), refractory epilepsy, and amyotrophic lateral sclerosis [75-79]. For instance, administration of *A. muciniphila* ameliorates AD-related pathologies by reducing cerebral amyloid-β deposition and enhancing cognitive performance in AD mouse models [77]. Clinical meta-analyses also support the relevance, revealing a significant depletion of *A. muciniphila* in the fecal microbiota of patients with ASD compared to that in neurotypical controls [76].

The therapeutic potential of *A. muciniphila* extends to seizure modulation; co-administration with *Parabacteroides merdae* reportedly exerts potent anti-seizure effects. Mechanistically, this intervention mimics the metabolic shifts induced by a ketogenic diet, reducing systemic gamma-glutamylated amino acids while elevating hippocampal γ-aminobutyric acid/glutamate ratios, thereby reinforcing the seizure threshold [75]. Furthermore, *A. muciniphila* abundance is positively correlated with the alleviation of cognitive dysfunction in HE, a neuropsychiatric syndrome, where it modulates the brain-derived neurotrophic factor and serotonin signaling pathways [80]. In FMT from humans into mice, the enrichment of *A. muciniphila* in the murine gut has been linked to superior spatial working memory [81].

While these associations are compelling, the precise molecular mechanisms by which *A. muciniphila* influences the CNS remain to be fully elucidated. It is hypothesized that these neuroprotective effects are primarily mediated through enhanced intestinal barrier integrity, which prevents the systemic translocation of pro-inflammatory endotoxins, and the optimization of metabolic homeostasis.

### Anti-Tumorigenic Immunity in Colorectal Cancer (CRC)

CRC remains a global health challenge, ranking second among malignancies in terms of mortality rate and exhibiting recurrence rates of up to 50% [82]. The initiation and progression of CRC are driven by the accumulation of somatic mutations in key tumor suppressor genes and oncogenes, such as transforming growth factor beta, tumor protein 53, Wingless-related integration site 1, and members of the B-cell lymphoma 2 family [83, 84]. Beyond genetic predispositions, accumulating evidence suggests that the gut microbiome acts as a critical rheostat for intestinal permeability and chronic inflammation, both of which are intrinsically linked to CRC pathogenesis [85-87]. Notably, *A. muciniphila* reportedly fortifies the intestinal barrier by augmenting mucin biosynthesis and tight junction integrity [48], while simultaneously stimulating cluster of differentiation (CD)8^+^ T cell-mediated immune responses to suppress colitis-associated tumorigenesis in murine models [52].

The resident gut microbiota significantly influences clinical outcomes in patients who undergo cancer immunotherapy, particularly with immune checkpoint blockade targeting the programmed cell death protein (PD)-1/programmed death ligand 1(PD-L1) axis. For instance, antibiotic treatment-induced gut dysbiosis is often associated with poor prognosis, a phenomenon that can be reversed by FMT from healthy donors [88, 89]. Mechanistically, administration of *A. muciniphila*-derived outer membrane vesicles or Amuc_1434 potentiates CD8^+^ T cell-mediated anti-tumor immunity through interaction with PD-L1 and increased levels of IFN-γ and IL-2 in CRC [90]. Furthermore, treatment with the C-terminal domain of Amuc_1100 (Amuc_C), a purified membrane protein from *A. muciniphila*, reportedly skews macrophage polarization from a pro-tumorigenic M2 phenotype toward an anti-tumorigenic M1 phenotype. This protein also recruits type 1 dendritic cells expressing major histocompatibility complex class II via TLR2 signaling, thereby inhibiting colorectal tumor growth [91].

Despite these beneficial attributes, recent evidence has introduced a more nuanced perspective regarding the role of *A. muciniphila* in CRC [92]. Paradoxically, certain studies have reported that high-dose administration of *A. muciniphila*, particularly following antibiotic-induced depletion of the native microbiota, may exacerbate CRC progression [93]. This detrimental effect supposedly results from a localized gut dysbiosis that increases intestinal permeability and paradoxically reduces mucin production, collectively fostering a pro-inflammatory milieu conducive to tumor expansion [94]. The dualistic role of *A. muciniphila* highlights the critical importance of dosage, timing, and the baseline composition of the host microbiota in determining therapeutic efficacy. Consequently, future research must focus on optimizing precision delivery strategies to maximize the on-target anti-tumor effects of *A. muciniphila* while mitigating potential systemic risks.

## *A. muciniphila* as Next-Generation Probiotics

*A. muciniphila* has emerged as a cornerstone of NGPs, demonstrating substantial therapeutic promise across a spectrum of host pathologies. The efficacy of live *A. muciniphila* is attributed to its active colonization of the gut mucosa, where it facilitates mucin turnover, produces SCFAs, and reinforces intestinal barrier integrity and immune modulation [25, 27, 30, 37, 45, 95]. However, recent paradigms have shifted toward using pasteurized *A. muciniphila* (typically heat-treated at 70°C), which has shown comparable or, in some contexts, superior efficacy. In preclinical models of DSS-induced colitis, pasteurized forms effectively alleviate colonic damage by enriching beneficial taxa, such as *Parasutterella* and *Dubosiella*, while simultaneously boosting sphingolipid metabolism and preserving tight junction integrity through the upregulation of claudin-1. Furthermore, emerging evidence extends the therapeutic reach of *A. muciniphila* to CRC via CD8^+^ T-cell-mediated anti-tumorigenic responses, neurodegenerative conditions through gut-brain axis modulation, and metabolic disorders by significantly increasing energy expenditure and reducing adiposity [48, 52, 69, 96].

The clinical viability of this approach was substantiated in a landmark human proof-of-concept trial [97]. Daily supplementation with 10^10^ pasteurized cells for 3 months in overweight and obese individuals (body mass index 27–40) was not only safe but also significantly improved metabolic health. Key findings included a reduction in insulinemia (–19.9%), lowered low-density lipoprotein-cholesterol (–8.3%), and decreased liver enzyme levels (gamma-glutamyl transferase –17.4%). Notably, the improvement in insulin sensitivity (+28.6% Matsuda ISI) surpassed that of both the placebo and comparable live probiotics. Combined with its favorable safety profile—highlighted by its approval as a novel food by the European Food Safety Authority and absence of adverse effects in high-dose 90-day rodent studies—pasteurized *A. muciniphila* represents a robust, practical, and potent candidate for a wide array of therapeutic indications.

## Bioactive Derivatives of *A. muciniphila* as Postbiotics

The therapeutic versatility of *A. muciniphila* is increasingly attributed to its diverse repertoire of bioactive derivatives, which function as potent molecular postbiotics. These components—spanning from structural membrane proteins and phospholipids to secreted enzymes, signaling peptides, and EVs—effectively recapitulate the multi-systemic benefits of the parent bacterium by engaging in precise molecular crosstalk with host cellular targets [37, 48, 55, 56, 98-100].

Rather than acting through a single pathway, these derivatives exert pleiotropic effects that go beyond simple immune modulation. Recent breakthroughs have revealed that *A. muciniphila*-derived effectors can directly orchestrate intestinal stem cell-mediated regeneration, drive epigenetic reprogramming in the tumor microenvironment, and even function as endogenous antagonists against systemic inflammatory sensors [101, 102].

**Fig. 1:**
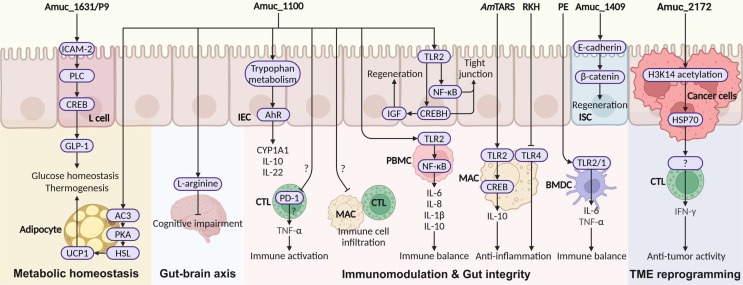
Multifaceted molecular mechanisms of *A. muciniphila*-derived bioactive derivatives in host metabolic and immunological homeostasis. Specific effectors derived from *A. muciniphila* (*e.g.*, Amuc_1631/P9, Amuc_1100, *Am*TARS, Amuc_1409, RKH, PE, Amuc_2172) orchestrate host physiology across four functional axes. (Metabolic homeostasis): Secreted P9 (Amuc_1631) engages the ICAM-2–PLC–CREB signaling axis in L cells to stimulate GLP-1 secretion, thereby improving systemic glucose homeostasis and thermogenesis. Amuc_1100 acts on adipocytes to promote thermogenesis via the AC3–PKA–HSL–UCP1 signaling axis leading to enhanced insulin sensitivity. (Gut-brain axis): Amuc_1100 rescues L-arginine levels to alleviate cognitive impairment. (Immunomodulation & Gut integrity): Amuc_1100 modulates AhR-targeted genes (*CYP1A1*, *IL10*, *IL22*) via tryptophan metabolism in IECs. Amuc_1100 activates CTLs by inducing TNF-α and downregulating PD-1. Amuc_1100 further suppresses immune cell infiltration through an undetermined signaling pathway (indicated by a question mark), while modulating immune homeostasis via the TLR2–NF-κB axis in PBMCs, resulting in the production of various cytokines (e.g., IL-6, IL-8, IL-1β, and IL-10). Amuc_1100 reinforces intestinal barrier functions by activating the TLR2–NF-κB and TLR2–CREBH pathways, the latter driving epithelial regeneration through IGF signaling. Crucially, secreted *Am*TARS specifically activates the TLR2–CREB axis to robustly induce IL-10 production in macrophages, while the tripeptide RKH functions as a TLR4 antagonist to provide protection against lethal sepsis. Additionally, a15:0-i15:0 PE tunes the immunological tone through TNF-α production by TLR2/1 heterodimer activation in BMDCs. Amuc_1409 interacts with E-cadherin to induce Wnt/β-catenin target genes, promoting ISC regeneration. (TME reprogramming): Amuc_2172 facilitates H3K14 acetylation and HSP70 secretion in colon cancer cells, reprogramming the tumor microenvironment toward an anti-tumor phenotype by activating CTLs to release IFN-γ. The question marks denote uncertainty regarding the proposed pathways. Abbreviations: IEC, intestinal epithelial cell; CTL, cytotoxic T lymphocyte; MAC, macrophage; PBMC, peripheral blood mononuclear cell; BMDC, bone marrow-derived dendritic cell; ISC, intestinal stem cell; ICAM-2, intercellular adhesion molecule-2; PLC, phospholipase C; CREB, cyclic AMP responsive element-binding protein; GLP-1, glucagon-like peptide-1; AC3, adenylate cyclase 3; PKA, protein kinase A; HSL, hormone-sensitive lipase; UCP1, uncoupling protein 1; AhR, aryl hydrocarbon receptor; CYP1A1, cytochrome P450 1A1; IL-10, interleukin-10; IL-22, interleukin-22; PD-1, programmed cell death protein-1; TNF-α, tumor necrosis factor-α, TLR2, Toll-like receptor 2; NF-κB, nuclear factor-κB; CREBH, cyclic AMP responsive element-binding protein H; IGF, insulin-like growth factor; IL-6, interleukin-6; IL-8, interleukin-8; IL-1β, interleukin-1β; TLR4, Toll-like receptor 4; HSP70, heat shock protein 70; IFN-γ, interferon-γ; TME, tumor microenvironment. Created with BioRender.com.

This section provides a comprehensive synthesis of how each distinct derivative—including the landmark Amuc_1100, the newly identified *A. muciniphila*-secreted threonyl-tRNA synthetase (*Am*TARS), and the metabolic regulator P9—modulates intestinal barrier reinforcement, systemic immunometabolic regulation, and anti-tumor immune responses. These interactions are systematically integrated into the mechanistic framework illustrated in Fig. 1, highlighting their potential as a new class of precision biotherapeutics.

### Structural Membrane Protein

Amuc_1100, a 34-kDa pili-like protein localized on the outer membrane of *A. muciniphila*, is the most extensively characterized bioactive derivative of the bacterium. A defining feature of Amuc_1100 is its remarkable thermostability, which preserves its functional integrity and therapeutic potency even after pasteurization. Its multifaceted roles were initially elucidated in the context of metabolic disorders, such as obesity and T2D [48]. In HFD-fed murine models, Amuc_1100 administration improved glucose tolerance and reduced both body weight and fat mass. Mechanistically, these metabolic improvements were driven by the upregulation of the adenylate cyclase 3 (AC3)-protein kinase A (PKA)-hormone-sensitive lipase (HSL) axis in adipocytes, which subsequently promotes lipolysis and UCP1-mediated thermogenesis [98].

Beyond metabolic regulation, Amuc_1100 acts as a pivotal mediator of intestinal homeostasis through its specific interaction with TLR2. This binding activates nuclear factor-κB (NF-κB) in epithelial cells, triggering the upregulation of key tight junction-associated genes, including cannabinoid receptor 1, claudin-3, and occludin. Furthermore, Amuc_1100 reinforces the gut barrier by inducing the expression of cyclic AMP-responsive element-binding protein H (CREBH), which upregulates protective tight junction proteins (claudin-5 and claudin-8) and suppresses inflammatory stress. Notably, CREBH also couples with the microRNA-143/145 cluster to positively modulate the insulin-like growth factor signaling pathway, thereby facilitating intestinal epithelial regeneration and mucosal wound repair [103].

The immunomodulatory capacity of Amuc_1100 is equally profound. It has been shown to attenuate colonic inflammation by suppressing the infiltration of macrophages and CTLs while upregulating aryl hydrocarbon receptor-targeted genes, including *CYP1A1*, *IL10*, and *IL22* [55]. In the context of colitis-associated CRC, Amuc_1100 paradoxically enhanced CTL activation and expansion through TNF-α induction and PD-1 downregulation, thereby exerting anti-tumorigenic effects [52]. These systemic immune-signaling capacities are further evidenced by its ability to modulate cytokine profiles (*e.g.*, IL-1β, IL-6, IL-8, IL-10) in human peripheral blood mononuclear cells (PBMCs) [49].

Recent studies have expanded the therapeutic scope of Amuc_1100 to the gut-brain axis. In aging models, Amuc_1100 ameliorates cognitive impairment by rescuing systemic L-arginine levels and fostering an environment enriched with L-arginine-producing commensals [104]. Moreover, it has demonstrated antidepressant-like effects in chronic unpredictable mild stress models, highlighting its potential as a neuropsychiatric intervention [105]. Collectively, the pleiotropic effects of Amuc_1100 across the metabolic, immunological, and neurological axes underscore its potential as a molecular biotherapeutic. However, its versatile functions necessitate further investigation into the intricate cross-talk between these pathways to fully harness its clinical applications.

### Secreted Signaling Proteins and Enzymes

Beyond its structural components, the therapeutic landscape of *A. muciniphila* has been significantly expanded by the identification of P9 (Amuc_1631), a novel 84-kDa secret protein. While previous studies consistently reported that *A. muciniphila* administration reduces body mass and decreases interscapular brown adipose tissue (iBAT) hypertrophy in HFD-fed mice, the specific molecular driver remained elusive until the characterization of P9 [37, 48].

Recent investigations have demonstrated that P9 serves as a potent metabolic rheostat by enhancing host thermogenic capacity. Specifically, P9 significantly induces the expression of uncoupling protein 1 (UCP1) and other key thermogenic differentiation markers in iBAT, thereby increasing functional metabolic expenditure [37].

The primary mechanism of action of P9 involves a sophisticated signaling axis within the gut-endocrine system. P9 directly interacts with ICAM-2 expressed on intestinal L-cells. This binding triggers the activation of phospholipase C and promotes intracellular Ca^2+^ signaling, which subsequently activates the CREB pathway. The culmination of this signaling cascade is the robust induction of GLP-1 secretion [37].

Furthermore, the metabolic benefits of P9 are complemented by its systemic immunomodulatory effects. P9 reduces systemic inflammation, a process partly mediated by the modulation of IL-6 levels [37]. By bridging gut-derived protein signaling with systemic glucose homeostasis and thermogenesis, the P9–ICAM-2 interaction represents a high-priority therapeutic target for the development of precision biotherapeutics against obesity and T2D.

The discovery of *Am*TARS by Kim *et al*. represents a transformative shift in the development of microbiome-derived therapeutics [56]. Beyond its biological role as a homeostatic mediator, *Am*TARS possesses several unique attributes that position it as a high-priority candidate for precision biotherapeutics targeting chronic inflammatory disorders.

Unlike conventional TLR2 ligands (*e.g.* Pam3CSK4) that predominantly trigger pro-inflammatory NF-κB signaling, *Am*TARS facilitates a selective activation of the TLR2–CREB axis [56]. This signaling bias is governed by its evolutionarily acquired unique regions (U1 and U2), which provide a discrete structural interface for TLR2 engagement. This interaction effectively orchestrates the mitogen-activated protein kinase (MAPK) and phosphatidylinositol 3-kinase-AKT pathways to phosphorylate CREB: the resulting activated CREB then outcompetes NF-κB for the limited pool of shared coactivator CREB binding protein/p300, thereby repressing pro-inflammatory cascades and tilting the immunological balance toward homeostasis.

The therapeutic significance of *Am*TARS is emphasized by its correlation with human disease states. Multi-omics analysis of IBD cohorts (IBDMDB) revealed that a reduction in *A. muciniphila*—and specifically its secreted *Am*TARS—is a hallmark of active ulcerative colitis and Crohn’s disease [56]. This suggests that *Am*TARS is an intrinsic immune checkpoint that, when depleted, leads to homeostatic collapse. In preclinical models, systemic administration of *Am*TARS showed therapeutic efficacy comparable to recombinant IL-10, significantly attenuating weight loss, colon shortening, and epithelial damage.

One of the most promising aspects of *Am*TARS for drug development is its compatibility with diverse delivery systems. Previous research has successfully demonstrated *Am*TARS can be delivered via engineered *Escherichia coli* Nissle 1917 [56]. This engineered probiotic effectively colonized the gut and secreted *Am*TARS *in situ*, where it targeted lamina propria macrophages to restore immune tolerance. This modularity—enabling both direct protein injection and live bacterial delivery—provides a scalable and versatile platform for treating not only IBD but also systemic inflammatory conditions, such as rheumatoid arthritis or lupus.

As an endogenous molecule produced by a core human commensal, *Am*TARS is evolutionarily tuned to the human immune system, potentially minimizing the immunogenicity and off-target effects often associated with synthetic drugs [56]. Its ability to maintain structural and functional integrity without a signal peptide further simplifies the bioengineering process for pharmaceutical production. Collectively, *Am*TARS defines a new class of commensal-derived biologics, offering a roadmap for utilizing the biological machinery of the microbiome to solve previously intractable inflammatory diseases.

While *A. muciniphila* has been extensively studied for its ability to ameliorate obesity-related metabolic diseases and colitis through EVs or secreted molecules, the direct enzymatic activity of commensal-derived protein on a host substrate remains largely unexplored. Recently, Jiang *et al*. identified Amuc_2172, an N-acetyltransferase specifically enriched in *A. muciniphila*–derived EVs rather than in the bacterial cytosol, as a potent mediator of anti-tumor immunity [101].

Amuc_2172 plays a pivotal role in reprogramming the tumor microenvironment by inducing the secretion of 70-kDa heat shock protein, which subsequently enhances tumor-specific CTL responses [101]. Remarkably, this bacterial enzyme engages in direct post-translational modification by promoting the acetylation of lysine 14 on histone H3 within CRC cells, thereby exerting epigenetic control over host gene expression.

To overcome the pharmacological limitations of systemic administration—where intraperitoneal injection of the purified protein fails to achieve sufficient intratumoral concentrations—a sophisticated delivery system utilizing bioengineered nanoparticles has been proposed [101]. This strategy ensures the targeted delivery of Amuc_2172 to the tumor microenvironment, maximizing its therapeutic efficacy in allograft mouse models. This study provides a rare and insightful example of how a microbiota-derived enzyme can directly modify host protein substrates, offering a new dimension to our understanding of the molecular crosstalk between commensal microbes and host immunity.

*A. muciniphila* has long been recognized for its role in promoting intestinal epithelial development, yet the specific microbial effectors governing this process remained largely uncharacterized until the recent identification of Amuc_1409. Using a comprehensive secretome profiling approach, Kang *et al*. [102] identified Amuc_1409 as a 16.5-kDa PepSY_like domain-containing protein and the most abundant constituent within the *A. muciniphila* secretome. Unlike structural components, this protein is actively secreted and possesses high thermal stability, enabling it to reach and communicate directly with host cells across the mucus barrier.

The primary physiological function of Amuc_1409 is to enhance intestinal stem cell (ISC) proliferation and regenerative capacity [102]. In both mouse and human intestinal organoids, treatment with Amuc_1409 significantly increased organoid size and lobe formation by upregulating markers of both active ISCs and differentiated cell lineages. The therapeutic potential of Amuc_1409 was further validated in several *in vivo* models of intestinal injury, including high-dose irradiation, chemotherapeutic drug (5-fluorouracil) exposure, and natural aging. In these models, oral administration of Amuc_1409 significantly delayed mortality, ameliorated weight loss and diarrhea, and accelerated mucosal repair by stimulating compensatory epithelial proliferation within the crypts.

Mechanistically, Amuc_1409 operates through a discrete structural interaction with the extracellular domain of E-cadherin [102]. This interaction promotes the internalization of E-cadherin, leading to the dissociation of the E-cadherin/β-catenin complex at the plasma membrane. Consequently, the released β-catenin translocates into the nucleus to activate the Wnt/β-catenin signaling pathway, which is indispensable for ISC self-renewal and lineage differentiation. Crucially, the effects of Amuc_1409 were abrogated in ISC-specific E-cadherin knockout models, confirming that E-cadherin is its essential host receptor. By restoring the functional decline of ISCs associated with injury and aging without promoting unlimited tumor proliferation, Amuc_1409 represents a potent molecular postbiotic and a promising biotherapeutic scaffold for enhancing intestinal resilience and treating gastrointestinal syndromes.

### Metabolite and Signaling Peptide

While the immunomodulatory roles of hydrophilic metabolites, such as SCFAs, are well-established in maintaining intestinal homeostasis [106], emerging evidence highlights the equal importance of microbiota-derived lipophilic metabolites. These lipid species contribute to immune tolerance by inducing Treg activity or modulating innate immune sensors [107]. For instance, α-galactosylceramides from *Bacteroides fragilis* have been identified as key regulators of colonic natural killer T cell activation in a structure-specific manner [108].

Building upon this framework, Bae *et al*. [99] recently identified a unique phospholipid, a15:0-i15:0 phosphatidylethanolamine (PE), derived from the cell membrane of *A. muciniphila*. This specific PE species functions as a ligand for the non-canonical TLR2–TLR1 heterodimer, mediating the bacterium’s distinctive immunomodulatory effects. Characteristically, a15:0-i15:0 PE is significantly less potent than classical, highly inflammatory TLR2 agonists (such as Pam3CSK4).

This low-affinity interaction is functionally significant; rather than triggering a robust pro-inflammatory burst, repeated low-level stimulation by this phospholipid appears to reset the immune activation threshold. By fine-tuning the responsiveness of the innate immune system, a15:0-i15:0 PE plays a crucial role in shaping the immunological tone of the gut, fostering an environment that is resilient to chronic inflammation while remaining vigilant against pathogens [99].

Gut microbiome-derived peptides (MDPs) have recently emerged as critical mediators of host-microbe crosstalk, providing protection against microbial infections and systemic inflammatory disorders. While many MDPs have traditionally been classified as antimicrobial peptides primarily involved in local pathogen inhibition, recent paradigms have identified specific MDPs that function as potent systemic immunomodulators. For instance, the lantibiotic nisin Z confers protection against bacterial infections by inducing chemokine secretion in human PBMCs [109].

In the context of life-threatening systemic inflammation, an innovative study by Xie *et al*. [100] identified a novel secreted tripeptide, RKH (Arg-Lys-His), specifically produced by live *A. muciniphila*. Clinical observations first revealed that the relative abundance of gut microbial *A. muciniphila* is significantly reduced in patients with sepsis compared with that in healthy controls, suggesting a protective role for this bacterium in sepsis pathogenesis. Subsequent metabolomics and validation in germ-free mice confirmed that live *A. muciniphila* produces RKH, which then enters the systemic circulation.

Mechanistically, RKH functions as a specific TLR4 antagonist, a rare feature among *Akkermansia*-derived components that more commonly engage TLR2 [100]. RKH directly binds to the TLR4 complex with high affinity (*K*_D_ = 3.34 nM), specifically interacting with functional sites on the TLR4 receptor. This binding competitively blocks the interaction between LPS and the TLR4/myeloid differentiation protein 2 complex, thereby thwarting the activation of pro-inflammatory signaling cascades (MAPK and NF-κB) and preventing the catastrophic cytokine storm.

The therapeutic efficacy of RKH has been rigorously substantiated in multiple preclinical models, including murine cecal ligation and puncture and piglet sepsis models, where its administration significantly improved survival rates and ameliorated multi-organ damage [100]. Notably, RKH treatment diminished the pro-inflammatory activity of macrophages from patients with sepsis without compromising T cell-immune functions, highlighting its potential as a safe and precision peptide-based biotherapeutic for acute systemic inflammatory conditions and endotoxemia.

### Perspectives and Future Directions

Since the landmark discovery of the *A. muciniphila* Muc^T^ strain, an extensive body of research has characterized the molecular conduits through which this bacterium interacts with host physiology to maintain intestinal homeostasis and ameliorate metabolic and inflammatory disorders [37, 48, 52, 56, 99, 102]. Advanced metagenomic sequencing and bioinformatic frameworks have further solidified the correlation between *A. muciniphila* abundance and disease resilience in both animal models and human cohorts. While its probiotic properties are well-documented, the field is now transitioning from describing general outcomes—such as barrier reinforcement and microbiota modulation—to elucidating target-specific molecular mechanisms. The restoration of gut barrier integrity is increasingly recognized as a master switch that sustains immune tolerance and facilitates precise multi-organ crosstalk between the gut and distal tissues, including the liver, adipose tissue, and the CNS. As a pioneering next-generation probiotic, *A. muciniphila* Muc^T^ is currently being evaluated in several clinical trials for metabolic syndrome, providing significant momentum for the development of microbiome-derived biotherapeutics [97].

Despite this promise, several critical factors must be addressed before *A. muciniphila* can be fully translated into clinical practice. First, its functional efficacy and colonization success are highly strain-specific [53]. Recent evidence indicates that clade-specific dynamics—such as the secretion of IgA by AmII (*A. muciniphila* clade II)-derived EVs that inhibits AmI (*A. muciniphila* clade I) colonization—govern the competitive advantages within the species [110]. These findings highlight the necessity of considering clade composition to optimize personalized microbiome therapies. Furthermore, the efficacy of *A. muciniphila* is fundamentally dictated by the host’s indigenous microbiota diversity and composition [111]. In certain pathological contexts, such as CRC progression, excessive mucin degradation could potentially exert adverse effects, highlighting the context-dependent nature of its therapeutic impact [112]. Therefore, comprehensive mechanistic studies within complex, individualized ecosystems are essential to ensure safety and efficacy.

Looking forward, the next frontier in microbiome medicine lies in the rational engineering of *A. muciniphila* strains. Strategies to enhance the production of specific bioactive molecules through clustered regularly interspaced palindromic repeats-Cas systems, phage-based engineering, or optimized culture conditions represent a shift toward precision biotherapeutics [113]. Such approaches enable the fine-tuning of key effectors—like *Am*TARS or RKH—while mitigating potential risks. However, significant challenges remain, including long-term safety concerns regarding genetically modified organisms, unforeseen ecological impacts on the commensal community, and technical hurdles of large-scale standardized manufacturing. Addressing these ethical, regulatory, and societal acceptance issues will be paramount in realizing the full potential of engineered *A. muciniphila* as a cornerstone of future precision medicine.

## Figures and Tables

**Table 1:** Overview of the physiological benefits and mechanisms of action of *A. muciniphila* in animal disease models.